# Editorial: Methods and applications in eating behavior

**DOI:** 10.3389/fpsyg.2022.1116363

**Published:** 2023-01-04

**Authors:** Michail Mantzios

**Affiliations:** Department of Psychology, School of Social Sciences, Birmingham City University, Birmingham, United Kingdom

**Keywords:** eating behavior and nutrition, eating behavior and obesity, eating behavior and eating disorder, eating behavior, methods

One of the confines in the field of eating behaviors is the lack of methods and applications to investigate avenues for the promotion of health. This series aimed to highlight the latest experimental techniques and methods used to investigate fundamental questions in Eating Behavior. This Topic considered technologies, interventions, practices, and up-to-date methods which will help advance scientific inquiry in the field.

Fischera et al. proposed a design on the interaction of norms and identity to form a new protocol for identifying fruit and vegetable consumption. Descriptive norms influenced fruit intake intentions and vegetable intake when investigated for the interaction with the identification manipulation. Nève et al. validated a visually aided dietary assessment tool, and all macronutrients and total energy intake were satisfactory estimations to capture dietary habits in older adults. The research adds an easier estimation of dietary habits and macronutrient intake. Zahedi et al. proposed methods for the effects of posthypnotic suggestions (PHS) on food-related decisions. While the hypotheses and the methods are detailed in the manuscript, the second stage of the manuscript is currently being reviewed. Javaras et al. developed the Cyberball-Milkshake Task, a method to facilitate research investigating individual differences in the consumption of highly palatable food due to ostracism. Initial findings propose a method that can be developed in assessing real-world behavior, and potentially propose a more pragmatic method of assessing experimentally aversive experiences and eating behaviors. Devonport et al. examined the effectiveness of two brief interventions (i.e., daily diaries and mindful eating practice + implementation intentions) aimed to help individuals deal with food cravings and associated emotional experiences during COVID-19. While the two interventions did not differ between them, the change in cravings and emotional states were significant and add to the support during times of needing remote interventions. Scoffier-Meriaux and Paquet examined the hypothesis of a Zone of Optimal Regulation of Eating Attitudes in Sport, where a detailed account of matching disordered eating attitudes and self-regulation of eating attitudes proposes a purpose and utility in sports psychology. Pristyna et al. proposed behavioral change dependent on personality characteristics, where they investigated the association between Big Five Personality Traits and nutrition-related variables. Of special interest were conscientiousness, extraversion, and neuroticism, which were associated with obesity. Chen et al. proposed two plant-based dietary indices to assess the association with diabetes, hypertension, and related chronic diseases, leading to the proposal of the development of dietary strategies to prevent illness and promote health. Susta et al. conducted a study that aimed to identify the differences in brain activity in participants with anorexia nervosa (vs. healthy controls) using visual stimulus conditions combined with Brain Activation Sequences. The implications of this method are evident, where both diagnostic and prognostic value is added to the timely utility of treatments and psychotherapy. Deng et al. proposed the efficacy and mechanism underlying intermittent fasting combined with lipidomic in male rats. Six hours of time-restricted feeding provided evidence of improvements in metabolic-associated fatty liver disease.

The research in this collection represents globally diverse authorship, with longitudinal, experimental, cross-sectional, and secondary data of new and devised methods to assess eating behaviors. The applicability across cultures and the modification for specific populations remain elements that require further research and collaboration. The results of this Research Topic are projected to aggregate the way that we think of and investigate eating behaviors and add to the literature on promoting health behavior change.

## Author contributions

The author confirms being the sole contributor of this work and has approved it for publication.

